# Status of Evidence on the Efficacy and Safety of Indian Traditional Medicine for Prediabetes and Type 2 Diabetes Mellitus: Protocol for a Systematic Review and Evidence Map Synthesis

**DOI:** 10.2196/70617

**Published:** 2026-04-30

**Authors:** Galib Ruknuddin, Azeem Ahmad, Manohar S Gundeti, Meena R, Eugene Wilson, Malik Itrat, Ghazala Javed, Mohd Aleemuddin Quamri, Deepti Singh Chalia, Pooja Yadav, Athul T P, Karthik K P, Anjana C S, Mythri H S, Aparna Dileep, Shifa Shetty P

**Affiliations:** 1 Ministry of Ayush, All India Institute of Ayurveda, Sarita Vihar, New Delhi, 110076, India, 91 8368960813; 2 Central Council for Research in Ayurvedic Science, New Delhi, India; 3 CCRAS-CARI, Mumbai, India; 4 Central Council for Research in Siddha, Chennai, India; 5 National Institute of Unani Medicine, Bengaluru, India; 6 Central Council for Research in Unani Medicine, New Delhi, India; 7 Central Council for Research in Homeopathy, New Delhi, India; 8 National Institute of Ayurveda, Haryana, India

**Keywords:** Ayurveda, Ayush systems, evidence mapping, homeopathy in diabetes, integrative medicine, naturopathy, prediabetes, quality of life, safety and efficacy, Siddha medicine, traditional medicine, type 2 diabetes mellitus, India, noncommunicable diseases, NCDs, Unani medicine, yoga-based interventions, Sowa Rigpa

## Abstract

**Background:**

Noncommunicable diseases, particularly diabetes, pose a growing global burden, with India disproportionately affected. India also has a rich repository of traditional medical systems—Ayurveda, yoga and naturopathy, Unani, Siddha, Sowa Rigpa, and homeopathy (AYUSH)—collectively governed under the Ministry of Ayush. These systems adopt a personalized and integrative approach to diabetes management, addressing glycemic control alongside metabolic and lifestyle factors. Despite growing use and evidence for AYUSH interventions, standardized evaluation methods remain limited.

**Objective:**

This study aims to quantitatively evaluate the evidence status for AYUSH interventions for the management of prediabetes and type 2 diabetes mellitus and establish a road map of evidence through research for better outcomes in the future.

**Methods:**

The systematic review will be conducted in accordance with the PRISMA (Preferred Reporting Items for Systematic Reviews and Meta-Analyses) guidelines and is registered in PROSPERO. All primary study designs, including randomized controlled trials, nonrandomized controlled trials, parallel-arm intervention trials, pretest-posttest trials, observational studies (including cross-sectional, case-control, and cohort studies), and case series and case reports will be assessed. Systematic reviews and meta-analyses will be screened for background information and identification of relevant primary studies but will not be included in the evidence synthesis. Studies involving AYUSH interventions either as stand-alone therapies or as an add-on to standard care will be reviewed. Electronic databases along with AYUSH-specific sources, including PubMed, CENTRAL, clinical trial registries, AYUSH Research Portal, MEDLINE, Scopus, Web of Science, Embase, Digital Helpline for Ayurveda Research Articles, and IndMED, will be searched using database-specific search strategies combining AYUSH-related and diabetes-specific keywords with Boolean operators. Outcome measures will include clinical recovery, biochemical parameters, quality of life, and adverse events. Data will be synthesized systematically and represented through an evidence map.

**Results:**

Database searches and pilot-testing of strategies are planned to commence in September 2025. Screening of eligible studies, data extraction, and quality assessment are planned for December 2025; data compilation and manuscript preparation will be conducted from July 2026 to October 2026; and the final systematic review and evidence map are anticipated by December 2026. Publication of the results is expected in early 2027. Anticipated findings will include a systematic integration of data relevant to the efficacy and safety profiles of AYUSH interventions for prediabetes and type 2 diabetes accompanied by an evidence map showcasing the allocation and reliability of the current evidence base on different AYUSH modalities.

**Conclusions:**

This review seeks to consolidate and evaluate existing data to facilitate evidence-based integration of Indian traditional medicine in diabetes management. The resulting evidence map will serve as a strategic tool for clinical research, health care policy, and future systematic reviews in the field of integrative medicine.

## Introduction

### Background

Diabetes mellitus (DM) is one of the world’s leading causes of morbidity and mortality, affecting individuals across all ethnicities, ages, and genders. It is characterized by persistently elevated blood glucose levels due to either the complete absence or inadequate production of insulin or the body’s inability to effectively use the insulin produced [1]. Type 2 DM (T2DM) is the most prevalent form, accounting for over 90% of DM cases worldwide [2]. In 2021, approximately half a billion people (537 million) were living with DM, a number projected to rise to 1.31 billion by 2050 [3].

India ranks among the top 4 countries with the highest number of DM cases alongside China, the United States, and Pakistan [4]. In 2021, India had 74.2 million people with DM, with projections indicating that this number will increase to 124.9 million by 2045. Moreover, India is second worldwide in the number of undiagnosed DM cases, with an estimated 39.4 million individuals (approximately 53.1%) unaware of their condition [5]. Prediabetes is defined by impaired glucose tolerance and impaired fasting glucose. In 2021, there were 464 million cases of impaired glucose tolerance worldwide; by 2045, that number is expected to rise to 638 million. Impaired fasting glucose was estimated to affect 5.8% of people worldwide in 2021 (298 million), and this number is expected to rise to 6.5% (414 million) by 2045 [6]. Although DM has become more treatable in recent decades, its morbidity and mortality rates still remain high, necessitating the development of new therapeutic strategies to combat the disease’s burden [7].

India’s traditional medical heritage is institutionalized under the Ministry of Ayush, that is, Ayurveda, yoga and naturopathy, Unani, Siddha, Sowa Rigpa, and homeopathy (AYUSH). These systems offer a comprehensive approach to health promotion and disease management through herbal formulations, dietary and lifestyle modifications, detoxification procedures, and mind-body practices. Given their wide-ranging scope and growing use in addressing noncommunicable diseases such as DM, a systematic evaluation of their efficacy and safety is essential—particularly for global readers who may be unfamiliar with these medical systems.

Over time, DM can cause life-threatening health problems, such as blindness, heart disease, kidney damage, and stroke [8]. Many patients with T2DM prefer not to use Western medicines due to the associated side effects, cost, and mode of administration (eg, injections) [9]. Previous systematic reviews of clinical trials suggest beneficial effects of several Ayurvedic medicines on T2DM-related outcomes, including improvement in blood glucose, with no major safety issues. However, one systematic review found that, among the included studies, the methodologies were not adequately reported, resulting in poorer methodological quality scoring [10]. Data from systematic reviews on the role of yoga suggest that it may lower oxidative stress and blood pressure; enhance pulmonary and autonomic function, mood, sleep, and quality of life; and reduce medication use in adults with T2DM. The study by Innes and Selfe also [11] cited limitations such as a lack of quantitative outcome data and a trial duration of less than 2 weeks.

DM-related medical expenses worldwide were predicted to reach US $966 billion in 2021 and are expected to rise to US $1054 billion by 2045 [12]. The expenses of treating the illness are rising in tandem with fatality rates. The average monthly expenditure per person for all of India is INR 1098.25 (US $11.88), which adds up to an annual expenditure per person of INR 13,179 (US $142.54) [13]. Traditional medicine systems may help reduce the economic burden from DM.

This study seeks to comprehensively assess the safety and efficacy of AYUSH interventions in the management of DM and prediabetes, aiming to identify existing gaps in current evidence and establish a dependable, evidence-based framework that supports informed decision-making and policy development for DM management.

### Objectives

The objectives of this review are as follows:

- To conduct a systematic quality assessment and synthesis of available evidence on the efficacy and safety of each Indian system of medicine (ie, AYUSH) for the management of prediabetes and T2DM
- To create an evidence map on the efficacy and safety of each Indian system of medicine for the management of prediabetes and T2DM to identify the gaps in available evidence for future research

## Methods

### Overview

The study has been framed using the population, concept, and context framework, focusing on individuals in India with prediabetes or T2DM, AYUSH interventions (both pharmacopoeial and nonpharmacopoeial), and studies conducted in Indian settings.

This systematic review will be conducted following the PRISMA (Preferred Reporting Items for Systematic Reviews and Meta-Analyses) guidelines [14]. The protocol has been registered with PROSPERO (registration number CRD42024545045).

### Study Design

This review will include primary study designs such as randomized controlled trials, nonrandomized trials, observational studies (including cross-sectional, case-control, and cohort studies), pretest-posttest designs, and case series and case reports conducted in India or involving Indian populations. Systematic reviews and meta-analyses will be consulted only for background information and identification of relevant primary studies and will not be included in the evidence synthesis (Table 1). Eligible studies must assess AYUSH interventions either as stand-alone therapies or as adjuncts to standard care for prediabetes and T2DM. Only studies published in English will be included. Eligible studies must report original interventional data on AYUSH management of prediabetes or T2DM, with clearly defined methodology, outcomes, and results.

**Table 1.** Inclusion and exclusion criteria.

Domain	Inclusion criteria	Exclusion criteria
Population	Human participants of any age or gender diagnosed with prediabetes or type 2 diabetes mellitus, including equivalent disease entities described in AYUSH^a^ systems (eg, “prameha,” “madhumeha,” “ziabetus” or “ziabetus shakri,” “neerizhivu noi,” “pramagham,” “madhumegham,” “innippu neer,” or “chin-sni”) with or without comorbidities	Animal studies, in vitro studies, or studies not involving human participants
Concept or intervention	AYUSH interventions used for the management of prediabetes or type 2 diabetes mellitus administered as stand-alone therapies or as adjuncts to standard or conventional care; includes pharmacopoeial (classic or proprietary formulations and “panchakarma”) and nonpharmacopoeial interventions (yoga, naturopathy, Siddha, Unani, Sowa Rigpa, homeopathy, and lifestyle and dietary modifications)	Interventions not related to AYUSH systems; studies not evaluating efficacy and/or safety outcomes
Comparator	Any comparator, including placebo, standard care, active comparator, or no comparator	—^b^
Outcomes	Studies reporting efficacy and/or safety outcomes, including biochemical, anthropometric, clinical, subjective, objective, quality of life, and adverse event data	Studies lacking clearly defined outcome measures or results
Study design	Primary research studies: RCTs^c^, nonrandomized trials, observational studies (cross-sectional, case-control, or cohort studies), pretest-posttest designs, case series, and case reports	Systematic reviews, meta-analyses, narrative reviews, editorials, commentaries, and conference abstracts without full text
Study setting and context	Studies conducted in India or involving Indian populations irrespective of setting (hospital, academic or research center, outpatient clinic, or community-based settings)	Studies conducted outside India without involvement of Indian populations
Language	Articles published in English	Articles published in languages other than English
Publication type	Published articles and eligible gray literature with accessible full text and sufficient methodological detail	Publications without full text and unpublished data without adequate methodological information

a AYUSH: Ayurveda, yoga and naturopathy, Unani, Siddha, Sowa Rigpa, and homeopathy.

b Not applicable.

c RCT: randomized controlled trial.

### Study Setting

Research carried out in India will be covered regardless of the type of setting—hospitals, academic or research centers, and outpatient clinics, among others.

### Type of Participants

Participants of any age and gender diagnosed with prediabetes or T2DM, or *prameha* or *madhumeha*, as defined in Ayurveda; *ziabetus* or *ziabetus shakri*, as defined in Unani; *neerizhivu noi*, *pramegham*, *madhumegha noi*, and *innippu neer,* as defined in Siddha; and *chin-sni*, as defined in Sowa Rigpa, with or without comorbidities will be included in this systematic review and evidence map generation.

### Sources

A comprehensive search will be conducted across scientific databases and AYUSH-specific sources: PubMed, CENTRAL, clinical trial registries, AYUSH Research Portal, MEDLINE, Scopus, Web of Science, Embase, Digital Helpline for Ayurveda Research Articles, and IndMED. A population, intervention, comparator, outcome, and study design (PICOS)–based strategy incorporating traditional medicine–related keywords will be used. Additionally, a gray literature search will be conducted through clinical trial registries (Clinical Trials Registry – India), institutional libraries, thesis repositories (Shodhganga), the Ayurvedic Research Database of the Institute of Teaching and Research in Ayurveda, Gujarat Ayurved University (Jamnagar, India), direct communication with study investigators, and relevant conference proceedings, ensuring a robust and inclusive evidence base. This approach ensures thorough inclusion of both published and gray literature.

### Search Strategy

A standardized data extraction form will be used to systematically capture all relevant data items, including demographic information of study participants, study characteristics, type of intervention, outcome measures, and quality assessment parameters. Additionally, a customized database-specific search strategy will be implemented incorporating relevant search terms formulated using the PICOS framework to ensure comprehensive and targeted retrieval of literature. The PubMed-specific search strategy shown in Table 2. Keywords such as “Madhumeha,” “Prameha,” “Ziabetus” and “Ziabetus Shakri,” “Neerizhivu noi,” “Pramagham,” “Madhumegham,” “Innippu Neer,” “Type II Diabetes,” “Prediabetes,” and “Diabetes Mellitus” will be combined using the appropriate Boolean operators “AND,” “OR,” and “NOR.” Further searches using the population, intervention, control or comparison, outcome, and study design framework will be conducted to narrow down the search results.

**Table 2.** Search strategy specific to PubMed.

Search set	Concept	Keywords or search terms	Boolean term
1	Ayurvedic or Indian disease terms	“Madhumeha” OR “Prameha” OR “Pramagham” OR “Madhumegham” OR “Neerizhivu noi” OR “Innippu Neer” OR “Ziabetus” OR “Ziabetus Shakri”	OR
2	Biomedical terminology	“Prediabetes” OR “Type II Diabetes” OR “Type-II Diabetes Mellitus” OR “Diabetes Mellitus”	OR
3	Combined disease terms	Search set 1 OR 2	OR
4	AYUSH^a^ or Indian traditional medicine	Ayurveda OR Siddha OR Unani OR Yoga OR Naturopathy OR AYUSH OR “Indian traditional medicine”	OR
5	Outcomes—efficacy	efficacy OR effectiveness OR management OR control OR treatment	OR
6	Outcomes—safety	safety OR toxicity OR adverse effects OR tolerability	OR
7	Outcome combination	Search set 5 OR 6	OR
Final search	Disease+AYUSH+outcomes	Search sets 3 AND 4 AND 7	AND

a AYUSH: Ayurveda, yoga and naturopathy, Unani, Siddha, Sowa Rigpa, and homeopathy.

### Interventions

All interventions from AYUSH systems used in the management of prediabetes and T2DM will be included in this review, whether administered as stand-alone therapies or as adjuncts to standard care. These interventions encompass both pharmacopoeial and nonpharmacopoeial approaches. Pharmacopoeial interventions include classic and proprietary formulations derived from herbal, herbo-mineral, or mineral origins, such as internal medicines and procedural therapies (ie, *panchakarma* in Ayurveda). Nonpharmacopoeial interventions comprise yoga practices (ie, *asana*, *pranayama*, and meditation), naturopathy procedures (ie, hydrotherapy and dietary regulation), Unani regimens, Siddha therapeutics, Sowa Rigpa treatment, homeopathic preparations, and system-specific lifestyle or dietary modifications. Each intervention will be evaluated with regard to formulation, dosage, and mode and duration of administration and assessed for efficacy and safety based on reported outcomes.

### Study Selection and Data Extraction

#### Overview

After implementing the prepared search strategy, 2 researchers will independently screen all the search results. Any disagreements in including the studies between them will be resolved through discussion with a third member. If disagreement persists, full texts will be retrieved and assessed in detail before a final eligibility decision is made. If additional clarification is required, the corresponding authors of potentially eligible studies will be contacted. Only articles published in the English language will be considered for inclusion. Eligible studies must meet predefined methodological standards. Systematic reviews and meta-analyses, if identified, will be consulted for background purposes only and will not be included in the evidence synthesis or quality assessment.

#### Data Extraction

A predesigned format will be used to extract all the relevant data items, including demographic details, year of publication, study design, efficacy, and safety outcome measure data, from the included studies for further analysis. AYUSH interventions will be collected in each specialty under 2 categories, stand-alone therapy and add-on to conventional or standard care, along with details of the control (if any) and duration of the study.

The outcome measures will be separately assessed for safety and efficacy, evaluating each for the subjective and objective measures. The biochemical, anthropometric, and quality of life data will also be extracted irrespective of time points. Safety data, including its adverse event data, and laboratory tests will be extracted. All the items required to assess the quality of the journals in which the included articles were published (eg, impact factor, CiteScore, SCImago Journal Rank, and indexing databases) will also be extracted.

### Outcome Measures

The outcome measures are stratified to distinctly assess prediabetes and T2DM, enabling structured synthesis and visual mapping of evidence. For prediabetes, efficacy will be evaluated using AYUSH-specific variables related to disease status and quality of life alongside objective clinical indicators, including fasting plasma glucose; 2-hour postprandial glucose; hemoglobin A_1c_; BMI; and insulin sensitivity or reserve, measured through homeostasis model assessment of insulin resistance and homeostasis model assessment of β-cell function. Safety outcomes will include the number and nature of reported adverse events, as well as abnormalities in lipid profiles, liver function tests, kidney function tests, or other biochemical markers cited in the included studies. For T2DM, outcome measures will encompass clinical recovery based on subjective and objective indicators, such as symptom resolution (as defined by AYUSH parameters), quality of life assessments, fasting plasma glucose, 2-hour postprandial glucose, hemoglobin A_1c_, BMI, homeostasis model assessment of insulin resistance, and homeostasis model assessment of β-cell function. Safety evaluations will similarly include the frequency of adverse events and relevant biochemical abnormalities.

### Quality Assessment of the Studies

The included studies will be subgrouped according to study design into randomized controlled trials, nonrandomized trials, observational cohort and cross-sectional studies, case-control studies, pretest-posttest designs, and case series involving human participants [15]. Quality assessment will be performed using study design–specific tools developed by the National Institutes of Health (NIH) and version 2 of the Cochrane risk-of-bias tool for randomized trials [16]. Systematic reviews and meta-analyses identified during the search will be consulted for background information and contextual understanding only and will not be included in the evidence synthesis or quality assessment; the AMSTAR (A Measurement Tool to Assess Systematic Reviews) checklist is referenced solely as a methodological standard for the appraisal of such secondary literature [17]. The quality of the journals where the included studies were published will be assessed based on metrics such as impact factor, SCImago Journal Rank, CiteScore, indexing databases, and Directory of Open Access Journals. Publication bias will be explored qualitatively by assessing selective outcome reporting, funding sources, and discrepancies between study protocols and published results as quantitative methods such as funnel plot analysis are not planned due to heterogeneity and lack of meta-analysis.

### Data Synthesis

The first objective entails systematically assessing and synthesizing evidence on the efficacy and safety of each Indian medical system, with data grouped by study type as outlined previously. The study quality assessment tools from the NIH will be used to evaluate the quality of the data collected. Appropriate tools will be selected based on study design, with each tool containing criteria that address aspects such as study population, group comparability, measurement methods, and statistical analysis. Reviewers will rate studies using these criteria, assigning responses such as “Yes,” “No,” or “Cannot determine,” which will then be translated into quality ratings of “Good,” “Fair,” or “Poor.” These ratings will be analyzed to determine the reliability and validity of the evidence, with higher-quality studies given more weight. The process and results will be documented to ensure transparency and reproducibility, thereby enhancing the rigor and credibility of the review.

For the generation of the evidence map for the efficacy and safety of AYUSH interventions for prediabetes and T2DM, we will begin with grading the evidence of safety and efficacy outcome measures from each study using the World Health Organization (WHO) grading system [18]. This involves categorizing studies into grade A (high quality), grade B (moderate quality), and grade C (low quality) based on criteria such as risk of bias, consistency, directness, and precision. Once graded, the evidence will be organized into a matrix with outcome measures in rows and intervention types (ie, AYUSH systems) in columns, further subcategorized by WHO grades. Each cell in this matrix will represent the number and quality of studies for a specific intervention-outcome pair. Analyzing the map will then involve identifying areas with high-quality evidence (multiple grade A studies), moderate- to low-quality evidence (grade B and C studies), and research gaps (few or no studies). Thus, we will be able to highlight areas of well-supported evidence and identify areas that need further investigation. The findings will be represented in tabular form, and we will also visualize them using charts that incorporate the results from both NIH and WHO tool analyses. We expect that these results will be helpful in clinical practice and future research. This approach will help us in presenting a clear, systematic picture of the evidence and, thus, will help in informed decision-making.

### Interpretation of Results

Outcomes and interventions in the included studies will be mapped into two subcategories: (1) AYUSH interventions for the management of T2DM and (2) AYUSH interventions for the management of prediabetes.

The obtained evidence will be further divided into 3 categories based on WHO criteria of grade of evidence for traditional and complementary medicine, and the quality of the evidence assessed based on the NIH tools will be represented using the colors green, yellow, and red. A tabulation of outcome measures separately into efficacy and safety under each of the traditional systems of medicine will be prepared for a graphical representation of the mapping of the evidence. Through this, we will attempt to identify the potential gaps in the existing literature and create a road map for future studies. This protocol will also help us understand the quality of research based on details of study design, sample, randomization, and quality of the journals it is published in, among other aspects.

## Results

The database search, along with screening and selection of eligible studies, is planned to commence in September 2025. Data extraction and quality assessment are planned for December 2025. Data compilation and manuscript preparation will be conducted from July to October 2026, with completion of the systematic review expected by December 2026. Publication of the results is expected in early 2027. This time frame may be revised based on the number of studies identified during the database search process and the execution of the analysis.

## Discussion

### Expected Findings

This protocol presents a systematic review and evidence map synthesis to interpret the efficacy and safety of Indian traditional medicine (AYUSH) for mitigating prediabetes and T2DM. The anticipated outcome is a comprehensive and narrative overview of the existing evidence landscape, showcasing robustly evaluated interventions such as herbo-mineral preparations; classic formulations; and integrative approaches, including yoga and lifestyle modifications, Unani formulations, Siddha therapeutics, and homeopathy remedies. The study is expected to identify the key areas, such as therapeutic effect and mechanism of action, of certain interventions, as well as evidence gaps, particularly related to safety profile, standardization, and the appropriateness of clinical trial design.

### Strengths and Limitations

This study will provide a comprehensive overview of AYUSH interventions for T2DM, which is an underexplored field in conventional reviews. The evidence map will act as a strategic tool to guide future research. The inclusion of clinical studies enables a multidimensional perspective of efficacy and safety.

Although the systematic review has strengths, it also has some limitations. The study is restricted to articles published in English and available through open access databases, which may result in the exclusion of relevant literature published in regional languages or subscription-based sources. Additionally, as the review focuses solely on studies conducted in India, the findings may not fully represent the global application of AYUSH interventions. The broad scope of this review and anticipated heterogeneity in study designs, outcome measures, and intervention types may pose challenges in direct comparison and uniform quality grading. Moreover, the methodological quality of many AYUSH studies may be variable, with some lacking sufficient rigor or standardized reporting. Reliance on metrics such as impact factor and indexing databases may favor English-language journals, potentially overlooking high-quality regional or nonindexed publications. Limited access to gray literature may also contribute to data gaps. The review is systematic in nature and does not include a meta-analysis, which may reduce the statistical strength of the overall conclusions.

### Conclusions

Generating robust evidence is crucial as we globally embrace traditional systems of medicine, addressing rising concerns about their safety and efficacy. Our community must prioritize the production of evidence-based data to establish the efficacy and safety of these treatments. By creating a map of the available evidence on traditional medicine, we facilitate better integration into mainstream health care systems and development of protocols that can be confidently applied to larger populations. This approach is particularly significant for managing major noncommunicable diseases such as DM, aligning with the Sustainable Development Goal of health for all. Moreover, thorough and systematic reviews will help reduce the prevalence of low-quality data, promoting a culture of publishing authentic and reliable research. Such evidence generation not only enhances the credibility of traditional medicine but also ensures its safe and effective use, ultimately contributing to a more holistic and inclusive global health framework. Through these efforts, we can better address health challenges, thereby improving overall public health outcomes and ensuring sustainable, long-term benefits for diverse populations worldwide.

AYUSH presents a promising option for managing the burden of DM as it emphasizes prevention over cure, offering better management of prediabetes to prevent progression to DM. Integrating yoga and personalized dietary guidelines, AYUSH provides a lifestyle approach that can significantly benefit individuals at risk of or managing DM. This personalized approach, coupled with preventive strategies, aligns well with global health goals and can effectively reduce the incidence and impact of DM, contributing to the overall reduction in the global disease burden.

Standard protocols for systematic reviews and evidence mapping are crucial as the authenticity of results depends on meticulously designed protocols that capture the right data and assess them accurately. These protocols ensure that the evidence generated is reliable and comprehensive, enhancing the credibility of traditional systems of medicine. By adhering to stringent standards, we can produce high-quality data that support the integration of traditional and modern medical practices, facilitating informed decision-making and effective health care strategies. This rigorous approach is essential for addressing safety and efficacy concerns, ultimately contributing to a more robust and trustworthy global health framework.

## Supplementary material

10.2196/70617 Checklist 1 PRISMA checklist.

## Abbreviations

AMSTAR: A Measurement Tool to Assess Systematic Reviews
AYUSH: Ayurveda, yoga and naturopathy, Unani, Siddha, Sowa Rigpa, and homeopathy
DM: diabetes mellitus
NIH: National Institutes of Health
PICO: population, intervention, comparator, and outcome
PRISMA: Preferred Reporting Items for Systematic Reviews and Meta-Analyses
T2DM: type 2 diabetes mellitus
WHO: World Health Organization
